# Effect of Binding Linkers on the Efficiency and Metabolite Profile of Biomimetic Reactions Catalyzed by Immobilized Metalloporphyrin

**DOI:** 10.3390/metabo12121269

**Published:** 2022-12-15

**Authors:** György T. Balogh, Balázs Decsi, Réka Krammer, Balázs Kenéz, Ferenc Ender, Tamás Hergert, Diána Balogh-Weiser

**Affiliations:** 1 Department of Chemical and Environmental Process Engineering, Budapest University of Technology and Economics, Műegyetem rkp. 3., H-1111 Budapest, Hungary; 2 Institute of Pharmacodynamics and Biopharmacy, Faculty of Pharmacy, University of Szeged, Eötvös u. 6, H-6720 Szeged, Hungary; 3 Department of Organic Chemistry and Technology, Budapest University of Technology and Economics, Műegyetem rkp. 3., H-1111 Budapest, Hungary; 4 SpinSplit LLC., Vend u. 17., H-1025 Budapest, Hungary; 5 Department of Electron Devices, Budapest University of Technology and Economics, Műegyetem rkp. 3., H-1111 Budapest, Hungary; 6 ThalesNano Ltd., Graphisoft Park, Záhony Str. 7, H-1031 Budapest, Hungary; 7 Department of Physical Chemistry and Materials Science, Budapest University of Technology and Economics, Műegyetem rkp. 3., H-1111 Budapest, Hungary

**Keywords:** biomimicking, drug metabolism, metalloporphyirin, chloroquine, immobilized catalyst, nanoporous carrier, continuous-flow process

## Abstract

The investigation of liver-related metabolic stability of a drug candidate is a widely used key strategy in early-stage drug discovery. Metalloporphyrin-based biomimetic catalysts are good and well-described models of the function of CyP450 in hepatocytes. In this research, the immobilization of an iron porphyrin was performed on nanoporous silica particles via ionic interactions. The effect of the metalloporphyrin binding linkers was investigated on the catalytic efficiency and the metabolic profile of chloroquine as a model drug. The length of the amino-substituted linkers affects the chloroquine conversion as well as the ratio of human major and minor metabolites. While testing the immobilized catalysts in the continuous-flow reactor, results showed that the presented biomimetic system could be a promising alternative for the early-stage investigation of drug metabolites regarding analytical or synthetic goals as well.

## 1. Introduction

A drug molecule administered in the human body usually undergoes different metabolic pathways catalyzed by a diverse repertoire of enzymes. These constitutive biotransformations typically start with an oxidative metabolic step mainly catalyzed by the cytochrome P450 (CyP450) isoenzyme family [1]. Accordingly, during the drug research and development phases, the metabolic stability of potential drug candidates against the CyP450 enzymes is one of the key information and is evaluated by using liver microsomes from different species as an industry gold standard in vitro test system. The importance of microsomal investigations is to predict the intrinsic hepatic clearance (Cl_int_) of the drug candidate and to evaluate the potential metabolites formed. The prior value shows how stable the drug molecule is to biotransformations in the liver and determines the oral dosage. Thus, the bioavailability of the drug and the latter is important to predict the easily attackable moieties on the molecule, which gives information for lead optimization to improve the structure for lower clearance and to assess the possible reactive metabolites formed [2,3].

The liver microsomal test systems are biologically relevant, and they provide adequate translational potency during the early phase of drug discovery; however, the biological origin and the internal properties of the system also carry several disadvantages. Usually, the substrate is used in limited concentration (≤10 µM), leading to a metabolite formation in small quantities. During the reaction, a complex, biological matrix is also present, not just because of the liver extract but also from the need for different coenzymes and cofactors (e.g., NADPH, NADP reductase, glucose-6-phosphate etc.). Based on these aspects, microsomal transformations mostly provide quantitative and limited qualitative information [4,5].

In recent decades, alternative solutions have been developed and aroused the interest of scientists to investigate methods that can replace or complement the CyP450-catalyzed oxidations [6]. Metalloporphyrins are important prosthetic groups in the hem proteins and enzymes of the living organism [7,8]. They are also present as an active site in the CyP450 enzymes in the form of protoporphyrin IX [9]. Based on structural similarity, synthetic metalloporphyrins can be used to mimic the monooxygenase or mixed-function oxidase activity of the CyP450 enzymes using organic peroxides or H_2_O_2_ as oxygen donors. Due to the specific catalytic activity of monooxygenase, metalloporphyrin-catalyzed oxidations can generally be considered biomimetic oxidative systems [10,11,12,13,14], which can also be supported by the fact that these systems could directly and robustly generate oxidative metabolites from the parent compound. However, metalloporphyrin analogues were successfully used for biomimetic reactions in many cases. Most synthetic metalloporphyrins are highly sensitive to oxidative medium in homogeneous reaction circumstances; thus, the catalyst degrades in the reaction, and as a result, its catalytic activity decreases irreversibly over time [15,16,17].

One option to improve the stability of metalloporphyrins by immobilization is using organic (e.g., Merrifield resin) or inorganic (e.g., silica or magnetic nanoparticle) supporters via covalent or secondary interactions like ionic bond [18,19,20]. The latter is an easy, fast and convenient method. However, the presence of ionizable functional groups (e.g., amino-, sulfonic acid- or carboxylic acid moiety) is required. Besides the stability improvement, the catalyst could be regained by a simple filtration, or it can be filled in a packed bed reactor and integrated into a continuous flow system. Surprisingly, despite the easy catalyst recovery and the possible high productivity, there are only a few examples in the field of continuous metabolite formation with the help of a flow chemical reactor [18,19].

Malaria, a tropical disease that can be spread by mosquitos, is nowadays still a life-threatening disease in the World. In 2020, more than 200 million cases were recorded, which resulted in more than 600,000 deaths [21]. According to historical notes, quinine bark has been used to treat malaria since the 17th century. The active ingredient quinine was isolated in 1820. However, the administration is still cumbersome, and several side effects have been recorded [22]. To mimic its structure, alternative drugs were developed, and among them, chloroquine was the first on the market, which was originally synthesized in Germany in 1934 by Hans Andersag and his coworkers at Bayer laboratories and marketed as Resochin^®^ [23]. Chloroquine (CQ) was used to treat malaria in higher quantities after World War II. However, its toxicity after long-term treatment and the appearance of chloroquine-resistant malaria plasmodium forced scientists to investigate novel structurally similar active agents. Shortly after the discovery of chloroquine, medicinal chemists started to develop alternative CQ analogues that dispose of superior properties. Among them, hydroxychloroquine (HCQ) was one of the most potent candidates with better tolerability, increased efficacy, and more favorable metabolism in the human body resulting in a better treatment against malaria [24]. However, next to malaria, CQ and HCQ are also effective in the treatment of Amebiasis [25] and Rheumatic diseases [26], and both have proven antiviral effects [27,28]. In 2004, CQ and HCQ were successfully used in the hospitalization of SARS [29]. As a result, during the emergence of serious SARS-CoV-2 (COVID-19) cases in the first half of 2020, the use of CQ and HCQ was the first among several other clinical treatments to alleviate the symptoms and accelerate the recovery process from the disease [30]. However, pre-clinical results were promising, but the efficacy of CQ or HCQ in preventing COVID-19 symptoms is not well evidenced. Considering potential safety issues, prophylaxis with CQ or HCQ against COVID-19 needs to be intensively evaluated in long-term clinical studies or high-quality randomized controlled studies [31,32,33].

Human metabolism of CQ is mainly related to the liver [34]. Despite the fact that the pharmacokinetic studies of the plasma samples of per os treated human voluntaries identified the formation of several potential metabolites, only the *N*-desethyl-chloroquine (DCQ) derivative formed by the main oxidative metabolic pathway had been identified in the in vitro human liver microsomal system [35]. However, both in vitro and in vivo pharmacokinetic studies analyze DCQ as the major CyP450-related metabolite of CQ, *N*-didesethyl-chloroquine (DDCQ) and the complete side chain dealkylation product 7-chloro-4-aminoquinoline as minor metabolites were also verified [36,37,38]. Other inconsistent in vivo minor metabolites are chloroquine side chain *N*-oxide and di-*N*-oxide derivatives [39].

**Figure 1:**
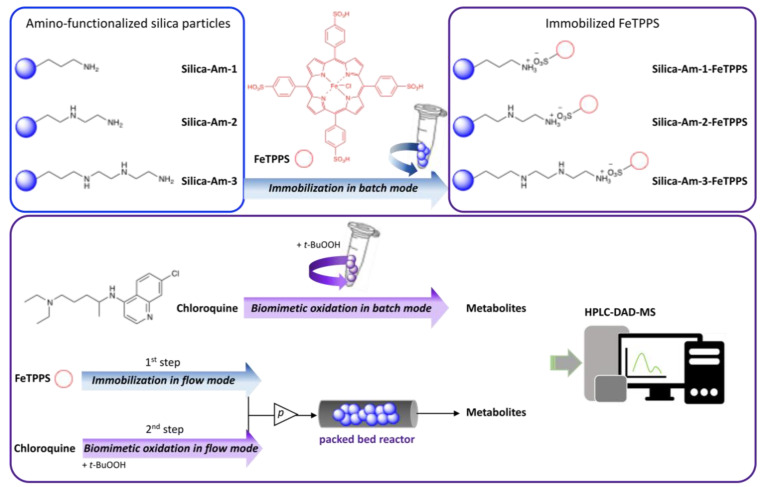
Immobilization of FeTPPS metalloporphyrin on silica particles functionalized with different amino-linker and application of immobilized FeTPPS for biomimetic oxidation of chloroquine in batch and continuous-flow mode.

Our previous work, in accordance with the literature, showed that metalloporphyirins are applicable biomimetic catalysts for the production of CyP450-like metabolites in several drugs with wide structural diversity. However, in the case of a metalloporphyrin-based biomimetic system, the stability, recovery, and sustainable applicability of the metalloporphyrin in continuous-flow reactors are important issues. Thus it requires immobilization using the proper solid carrier (Figure 1) [19,20]. In this paper, we report a simple and facile formation of a biomimetic system using an optimized solid carrier, which is applicable in a packed bed continuous-flow reactor. The aim of this work was to investigate the catalytic activity of metalloporphyrin by its ionic immobilization to the silica support modified with an aminopropyl side chain and, possibly, the regioselectivity of the biomimetic oxidation by changing the length of the aliphatic primary amine linker. In the case of chloroquine, chosen as a well-known model compound, the aim was to investigate whether the product ratio of the major dealkylation metabolites and the efficiency of the substrate transformation can be influenced. The stability of the metalloporphyrin provided by the immobilization system, as well as its loading into a packed-bed reactor, enabled next to the usual batch tests to investigate the biomimetic conversion of chloroquine also in a continuous-flow system that is orders of magnitude more efficient.

## 2. Materials and Methods

### 2.1. Materials

All solvents used in this experiment were of analytical grade. Methanol (MeOH), ethanol (EtOH), trifluoracetic acid (TFA), acetic acid, *N*-(2-aminoethyl)-3-aminopropyl-trimethoxysilane (Am-2) were purchased from Merck Ltd. (Budapest, Hungary). Water was obtained from a Millipore (Bedford, MA, USA) Milli-Q water-purification system and applied for the preparation of all aqueous solutions. Chloroquine and *t*-butyl hydroperoxide (*t*-BuOOH), sodium-acetate × 3 H_2_O, 3-aminopropyltrimethoxysilane (Am-1), *N*^1^-[3-(trimethoxysilyl)propyl]-diethylene-triamine (Am-3) were purchased from Sigma-Aldrich (St. Louis, MO, USA). 5,10,15,20-tetrakis-(4-sulfonatophenyl)iron(II) porphyrin (FeTPPS) was purchased from Frontier Scientific (Logan, UT, USA). Aqueous ammonium hydroxide solution (25%) was purchased from Fisher Scientific (Waltham, MA, USA). Davisil^®^ 250 [40–63 µm] (Dv250) silica gel was purchased from W. R. Grace & Co. (Deerfield, IL, USA). Human (M1000: Human-Male), mouse (M1000: Mouse-CD1) and rat (R1000: Rat-Sprague Dawley) liver microsomes were purchased from Xenotech Llc. (Kansas City, MI, USA).

### 2.2. Methods

#### 2.2.1. Metabolism of Chloroquine by Human, Rat, and Mouse Microsomal Reaction

Sodium pyrophosphate (125 µL, 6.38 mg mL^−1^), magnesium chloride (50 µL, 3 mg mL^−1^), glucose-6-phosphate (25 µL, 13 mg mL^−1^), glucose-6-phosphate dehydrogenase (25 µL, 20 IU mL^−1^), TrisHCl buffer (170 µL, 15.76 mg mL^−1^), human, rat and mouse liver microsome (50 µL, final concentration is 1000 µg mL^−1^) and chloroquine solution (in methanol, 5 µL, 0.65 mg mL^−1^) was pipetted in an Eppendorf tube, then was held at 37 °C for 5 min. After that, NADPH (50 µL, 3.72 mg mL^−1^) was added to the reaction mixture. It was shaken for 30 min at 37 °C using an orbital tube shaker (ThermoMixer, Eppendorf, ThermoScientific, Waltham, WA, USA). The mixture was quenched with methanol (0.5 mL, −20 °C). The microsomes were separated by ultracentrifugation (at 10,000× *g*, for 5 min, Micro CL 17, ThermoScientific Inc., Waltham, MA, USA). The clear upper phase (0.8 mL) was analyzed by the LC-DAD-MS method described in Section 2.2.2.

#### 2.2.2. HPLC-DAD-MS Measurement

The component analysis of reaction media was carried out on an Agilent 1200 high-pressure liquid chromatography system (HPLC) with a diode array detector (DAD) coupled with a 6410 QQQ-MS (Agilent Technologies, Palo Alto, CA, USA), supported with a vacuum degasser, a binary pump, mixer assembly, an autosampler, a column temperature controller at 45 °C. Kinetex EVO C18 column (50 × 3 mm, 2.6 µm) (Phenomenex, Torrance, CA, USA) was applied as the separation phase, flow rate of 1.45 mL min^−1^ was used for the mobile phase. The composition of eluent A was 0.1% (*v*/*v*) trifluoroacetic acid (TFA) in water (pH 1.9), eluent B was a mixture of acetonitrile and water in 95:5 (*v*/*v*) with 0.1% (*v*/*v*) TFA. A linear gradient of 2–100% B was applied at a range of 0–4.9 min, then 100% B at 4.9–6.0 min. It was followed by a 1.20 min equilibration period prior to the next injection with volume 5 µL. Chromatograms were registered at wavelength 220 ± 4 nm. The MSD operation was performed as follows: ESI positive ionization, scan ion mode (100–900 *m*/*z*), drying gas temperature 350 °C, nitrogen flow rate 11 L min^−1^, nebulizer pressure 40 psi, quadrupole temperature 100 °C, capillary voltage 4000 V, fragmentor voltage 135 V. The data was collected and evaluated by MassHunter B.01.03 software (Santa Clara, CA, US). Representative LC-DAD chromatograms and MS spectra of chloroquine and its metabolites are provided in the SI.

#### 2.2.3. Biomimetic Oxidation of Chloroquine Catalyzed by Dissolved FeTPPS Metalloporphyrin

Chloroquine solution (50 µL, 4.55 mg mL^−1^ in methanol:sodium acetate buffer, 4:1 *v*/*v*, pH = 4.5, 64 mM), solvent completion (150 µL, methanol:sodium acetate buffer, 4:1 *v*/*v*, pH = 4.5), porphyrin solution (50 µL, 0.9 mg/mL in methanol:sodium acetate buffer, 4:1 *v*/*v*, pH = 4.5, 64 mM) and oxidizing agent solution (*t*-BuOOH, 50 µL, 88.2 mM in methanol:sodium acetate buffer, 4:1 *v*/*v*, pH = 4.5, 64 mM) was pipetted in an Eppendorf tube. It was shacked for 1 h at room temperature at 400 rpm. The reaction mixture (300 µL) was analyzed by the LC-DAD-MS method described in Section 2.2.2.

#### 2.2.4. Surface Functionalization of Silica Particles

Silica particles (4.0 g) were shaken for 1 h in a mixture of ethanol (40 mL) and an aqueous solution of ammonium hydroxide (640 µL, 25%). After 1 h the corresponding organosilane reagent (200 µL, Am-1, Am-2 or Am-3) was added, then the suspensions were shaken (room temperature, 450 rpm, orbital shaker Vibramax 100, Heidolph NA Llc., Schwabach, Germany) for 24 h, then further organosilane was added (600 µL), and it was shaken for further 48 h. Silica particles were washed with ethanol (3 × 10 mL) using centrifugation (15 min, 10,000 rpm, 5 °C, Z300K, Hermle AG, Gosheim, Germany). The functionalized silica particles were dried on open-air until constant mass.

#### 2.2.5. Immobilization of FeTPPS Metalloporphyrin on Functionalized Silica Particles in Batch Mode

The corresponding silica particles (200 mg, Silica-Am-1, Silica-Am-2 or Silica-Am-3) were measured in a centrifuge tube, then FeTPPS solution (5 mL, 0.9 mg mL^−1^, MeOH) was added, and the suspension was shaken for 5 min (room temperature, 450 rpm, orbital shaker Vibramax 100, Heidolph NA Llc., Schwabach, Germany). FeTPPS bounded silica particles were centrifuged, and a sample (900 μL) was taken from the supernatant for the determination of immobilization yield (see in Section 2.2.6) and then washed with methanol (3 × 10 mL) using centrifugation (15 min, 10,000 rpm, 5 °C, Z300K, Hermle AG, Gosheim, Germany). The FeTPPS bounded silica particles were dried on open-air until constant mass.

#### 2.2.6. Determination of Immobilization Yield (Y_I_)

After the immobilization of FeTPPS metalloporphyrin, a sample (900 μL) was taken directly from the residual binding solvent from both batch and continuous flow mode as well, and it was analyzed by Genesys type 2 UV-VIS spectrophotometer (Thermo Fisher Scientific Inc., Waltham, MA, USA) at room temperature. The specific wavelength (λ_max_) of FeTPPS was determined (λ_max_ = 395 nm), then calibration curves were also recorded. Immobilization yield (*Y*_I_, %) was calculated as follows:

(1) $$Y_{I}=\frac{c_{2P}}{c_{1P}}\times 100$$

where c_1*P*_ is the initial porphyrin concentration, and c_2P_ is the residual porphyrin concentration in the binding solution.

#### 2.2.7. SEM/EDAX Analysis

The surface elemental composition of supported metalloporphyrin samples was analyzed by JEOL JSM-5500LV scanning electron microscope (SEM), and the element analysis was carried out with energy dispersive spectroscopy/energy dispersive X-ray analysis (EDS/EDAX with Si(Li) detector) applying 20 kV accelerating voltage and sampling time of 60 s. Measurements were performed in triplicate.

#### 2.2.8. Biomimetic Oxidation of Chloroquine Catalyzed by Dissolved FeTPPS in Batch Reaction Mode

In an Eppendorf tube chloroquine solution (50 µL, 4.55 mg mL^−1^ in methanol:sodium acetate buffer, 4:1 *v*/*v*, pH = 4.5, 64 mM), solvent completion (150 µL, methanol:sodium acetate buffer, 4:1 *v*/*v*, pH = 4.5), porphyrin solution (50 µL, 0.9 mg mL^−1^ in methanol:sodium acetate buffer, 4:1 *v*/*v*, pH = 4.5, 64 mM) and oxidizing agent solution (*t-*BuOOH, 50 µL, 88.2 mM in methanol:sodium acetate buffer, 4:1 *v*/*v*, pH = 4.5, 64 mM) were shaken. It was shaken for 1 h at room temperature at 400 rpm using an orbital tube shaker (ThermoMixer, Eppendorf, ThermoScientific, Waltham, MA, USA). The reaction mixture was analyzed by the LC-DAD-MS method described in Section 2.2.2.

#### 2.2.9. Biomimetic Oxidation of Chloroquine Catalyzed by Immobilized FeTPPS in Batch Mode

In Eppendorf tubes, porphyrin loaded silica carrier (2.0 mg) and chloroquine solution (0.45 mL, 0.5 mg mL^−1^ in methanol:sodium acetate buffer 4:1 *v*/*v*, pH = 4.5, 64 mM) were sonicated for 20 min using an ultrasonic bath (Sonorex Digitec DT31, Bandelin, Berlin, Germany). The reaction was started with the addition of the oxidizing agent (*t*-BuOOH, 50 µL, 88.2 mM in methanol:sodium acetate buffer, 4:1 *v*/*v*, pH = 4.5, 64 mM), then it was shaken for 1 h at room temperature at 400 rpm using an orbital tube shaker (ThermoMixer, Eppendorf, ThermoScientific, Waltham, MA, USA). The reaction mixture was analyzed by the LC-DAD-MS method described in Section 2.2.2.

#### 2.2.10. Immobilization of FeTPPS Metalloporphyrin on Functionalized Silica Particles in Continuous-Flow Mode

The corresponding silica particles (Silica-Am-1, Silica-Am-2 or Silica-Am-3) were put inside a stainless steel CatCart^®^ column (length 24 mm, inner diameter 4 mm, outer diameter 5 mm, ThalesNano Inc., Budapest, Hungary), then it was integrated into a Phoenix^TM^ Flow Reactor (ThalesNano Inc., Budapest, Hungary), the prefilled column was washed with an aqueous acetic acid solution (2 mL, 10%) next to 0.5 mL min^−1^ flow rate then FeTPPS solution (5.0 mL, 0.9 mg mL^−1^ in MeOH) was pumped through with rate 0.5 mL min^−1^. The bed was washed with the mixture of methanol:sodium acetate buffer (20 mL, 4:1 *v*/*v*, pH = 4.5, 64 mM, flowrate 0.5 mL min^−1^) and the sample was taken for the determination of immobilization yield (see in Section 2.2.6).

#### 2.2.11. Biomimetic Oxidation of Chloroquine Catalyzed by Immobilized FeTPPS in Continuous-Flow Mode

Chloroquine solution (0.5 mg mL^−1^, in MeOH:sodium acetate buffer-4:1 *v*/*v*, pH 4.5, 64 mM) containing oxidizing agent (*t*-BuOOH, 2 molar equivalents) was pumped through the reactor with a flow rate of 0.25 mL min^−1^ (see in Section 2.2.10), the sample was taken after 20 min for 5 min and was analyzed by HPLC-DAD-MS described in Section 2.2.2.

#### 2.2.12. Calculation of Biomimetic Reaction Parameters

The conversion of the substrate (*c*), turnover number (TON) and space-time yield (STY, mg L^−1^ h^−1^) were calculated by using the following equations based on HPLC chromatograms:

(2) $$c\;\left[\%\right]=\left(\frac{n_{P}}{n_{S}+n_{P}}\right)\times 100$$

where *n_S_* and *n_P_* are the molar amounts of the substrate (*S*) and product (s) (*P*),

(3) $$TON\left[–\right]=\frac{n_{P}}{n_{cat}}$$

where *n_P_* and *n_cat_* are the molar amounts of product (s) (*P*) and catalyst (*cat*),

(4) $$STY\left[mg\;l^{-1}h^{-1}\right]=\frac{m_{p}}{V_{r}\times t}$$

where *m_p_* is the mass of the products in mg, *V_r_* is the volume of the reactor and *t* is time.

## 3. Results

### 3.1. Metabolic Stability Study for Chloroquine by Liver Microsome from Different Origins

**Figure 2:**
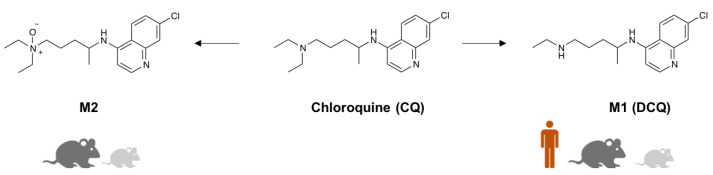
In vitro metabolite profiles of chloroquine (CQ) based on human, rat and mouse liver microsomal investigation. **M1**: N-desethyl-chloroquine (DCQ), **M2**: N-oxide derivatives of chloroquine.

Hepatocyte-derived microsomal tests as a gold standard for determination of the metabolic stability of biologically active molecules were performed applying human, rat and mouse microsomes (HLM: human liver microsome, RLM: rat liver microsome and MLM: mouse liver microsome) since they are commonly applied in early stage investigations. The metabolites of chloroquine (CQ) as model substrate were identified by LC-DAD-MS and the substrate conversion was also calculated. Results showed, accordingly to the literature, that human, rat and mouse liver microsome produced **M1** (N-desethyl-chloroquine, DCQ) as a major metabolite, rat and mice also provided **M2** as a minor metabolite (N-oxide derivative of CQ). Similar to the results of a previous human microsomal study [32], **M3** (didesethyl-chloroquine, DDCQ), which is also in vivo minor metabolite in human origin, was not identifiable in our in vitro investigations (Figure 2).

Based on LC-DAD-MS measurements, the productivity of microsomal systems was not really high, while the conversion of Chloroquine was only cc. 2–5% (Table 1). It is not an unexpected result because, after single-dose treatment of human patients, CQ was detectable in their blood and urine for more than seven and seventeen weeks, respectively [34]. Thus, the increased stability of chloroquine obtained in our microsomal study is well-compatible with the extremely slow in vivo clearance. **M1** (CQ − 28u) deethylation metabolite was the major component for each three liver microsomes; **M2** (CQ + 16u) mono-oxidation metabolite can be detected with the highest value in mice, which was the most active in vitro system, regardless.

### 3.2. Immobilization of Metalloporphyirin onto Amino-Functionalized Silica Particles

**Figure 3:**
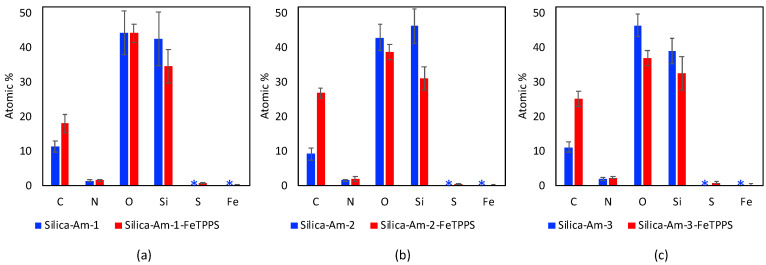
Elemental analysis of amino-functionalized silica particles ((**a**) Silica-Am-1, (**b**) Silica-Am-2 and (**c**) Silica-Am-3 marked with 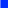) and FeTPPs immobilized onto amino-functionalized silica particles ((**a**) Silica-Am-1-FeTPPS, (**b**) Silica-Am-2-FeTPPS and (**c**) Silica-Am-3-FeTPPS marked with 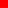) by SEM-EDAX. * atomic% < 0.1%).

Irregular nanoporous silica particles with pore size 25 nm and diameter 40–60 µm were modified with trimethoxysilanes substituted by primer amino function groups (Am-1: 3-aminopropyltrimethoxysilane, Am-2: 3-aminoethyl-aminopropyltrimethoxysilane, Am-3: N^1^-[3-(trimethoxysilyl)propyl]-diethylene-triamine) for ionic binding of FeTPPS (5,10,15,20-tetrakis-(4-sulfonatophenyl)iron(II) porphyrin). To monitor the efficacy of the FeTPPS immobilization, residual binding buffers were investigated by UV-Vis spectroscopy (according as described in Section 2.2.6). Based on the characteristic absorption spectra of FeTPPS, the immobilization yield (Y_I_) for Silica-Am-1, Silica-Am-2 and Silica-Am-3 carriers was 100%. Thus there were no detectable FeTPPS in the residual binding solution. However, in the case of silica particles without an amino-function group as blank sample, Y_I_ was <0.1%. The immobilized FeTPPS (Silica-Am-1-FeTPPS, Silica-Am-2-FeTPPS and Silia-Am2-FeTPPS) catalysts and amino-functionalized silica particles (Silica-Am-1, Silica-Am-2 and Silica-Am-3) were characterized by SEM coupled EDX (Energy Dispersive X-ray analysis) (Figure 3). Regarding the atomic content (Atomic%) of the carriers, the presence of FeTPPS on silica particles can be obviously detected, while the C, S and Fe content of immobilized samples was significantly higher (C: 18.1 ± 2.6%, S: 0.9 ± 0.2%, Fe: 0.4 ± 0.1%; C: 26.9 ± 1.6%, S: 0.7 ± 0.1%, Fe: 0.3 ± 0.1%; C: 25.3 ± 2.3%, S: 1.0 ± 0.2%, Fe: 0.4 ± 0.2% for Silica-Am-1, Am-2 and Am-3 respectively) then the naked, amino-functionalized silica carriers (C: 11.4 ± 1.6%, C: 9.3 ± 1.7% and C: 11.3 ± 1.4% for Silica-Am-1, Am-2 and Am-3 respectively and atomic% of S and Fe elements were under 0.1% for each cases). In summary, according to the results of two independent analysis (UV-Vis spectroscopy and SEM-EDX), it can be said that the immobilization of FeTPPS on amino-functionalized silica particles was successful in each case of amino-linker (Am-1, Am-2 and Am-3).

### 3.3. Biomimetic Oxidation of Chloroquine by Dissolved and Immobilized Metalloporphyrin in Batch Mode

**Figure 4:**
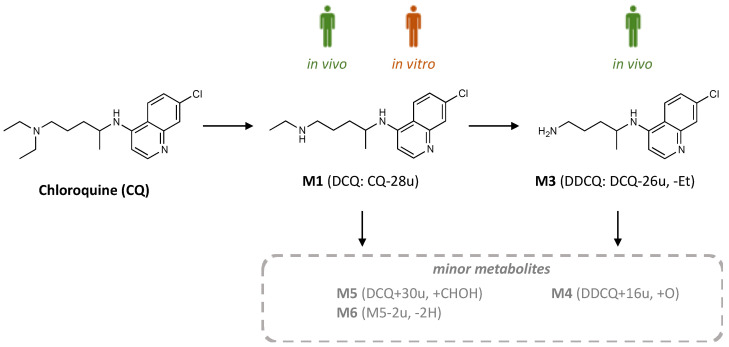
Metabolites formation pathway of chloroquine (CQ) based on FeTPPS metalloporphyrin catalyzed biomimetic oxidation. **M1**: N-desethyl-chloroquine (DCQ), **M3**: N-didesethyl-chloroquine (DDCQ), and minor metabolites **M4**, **M5**: hydroxymethylated N-desethyl-chloroquine and **M6**: N-formamide analogue of **M5**.

Biomimetic oxidation of chloroquine (CQ) was performed applying FeTPPS metalloporphyrin in its dissolved and immobilized forms in a simple shaked vial, batch mode. The reaction media were investigated by HPLC-DAD-MS, conversion of CQ and metabolite contents were also determined (Figure 4 and Table 2).

Homogenous FeTPPS solution and immobilized FeTPPS on silica particles worked with higher catalytic efficiency than microsomal systems, while conversion values were cc. 18–50% for each case. However, the highest CQ conversion could be achieved by FeTPPS immobilized on Silica-Am-1 carrier. Regarding the metabolic patterns, novel biomimetic metabolites can also be identified with the FeTPPS-based catalyst. Next to the major **M1** (deethylation), **M3** (dideethylation) as the minor human in vivo metabolite can be found at remarkably amount in the reaction mixtures catalyzed by FeTPPS bounded on Silica-Am-1 and Silica-Am-2. **M4** as minor and M5, M6 as artificial minor biomimetic metabolites can be also observed (**M4**: mono-oxidation product of **M3** with dissolved FeTPPS and all of the immobilized forms, **M5**: hydroxymethylated derivative of **M1** with only dissolved FeTPPS and **M6**: oxidative dehydrogenation of **M5** with dissolved and immobilized FeTPPS onto Silica-Am-1 carrier) in a small amount (0.1–0.2%). Other trace biomimetic metabolites without exact MS data can be detected in the case of dissolved FeTPPS and immobilized on Silica-Am-1 particles. Results showed that the FeTPPS binding amino-linker can influence the catalytic activity of the biomimetic system, while the shorter the length of amino-linker, the higher the conversion could be achieved. One should notice, that FeTPPS immobilized on silica with longer linker chains (Silica-Am-2 and Silica-Am-3) gave fewer metabolites then dissolved or immobilized FeTPPS on the carrier with the shortest amino-function group (Silica-Am-1).

### 3.4. Biomimetic Oxidation of Chloroquine by Dissolved and Immobilized Metalloporphyirin in Continuos-Flow Mode

The immobilization of metalloporphyrin onto solid carrier with proper morphology allows the application of biomimetic reactions in continuous-flow mode. FeTPPS metalloporphyrin bounded to silica particles was integrated into a packed-bed flow reactor, then the substrate containing reaction media was let through the system. Samples from the output were collected and analyzed with LC-DAD-MS (Table 3). This study obviously presented that silica-bounded metalloporphyrins could be successfully applied in a continuous flow process and the effect of binding linker can also be realized as in the case of simple batch systems. Regarding the conversion values, only cc. 2% conversion was observed with silica modified by the shortest linker (Silica-Am-1), the longer amino-linker gave better results, especially silica with medium linker length (Silica-Am-2) provided the highest catalytic effectivity, while conversion of chloroquine was cc. 76%. In addition, only **M1** and **M3** metabolites were formed in continuous-flow systems next to other side products. To conclude these results, silica carrier with the longest binding chain can provide a really effective FeTPPS catalysis, while conversion value was more than 50% and only a few amount (cc. 2%) side product was detectable.

### 3.5. Comparison of the Effectivity of Liver Microsomal and Biomimetic Systems for the Investigation on Chloroquine Metabolism

For better comparison of different FeTPPS-based biomimetic and microsomal systems, Turn Over Number (TON) and Space Time Yield (STY) were determined (Table 4). TON can be added for only FeTPPS containing reactions, while the exact CyP450 related metalloporphyrin content of the microsome is unknown. TON-s were similar with each FeTPPS catalyst in batch mode, thus the immobilization of FeTPPS did not degrade the catalytic properties of the metalloporphyrin. TON in continuous-flow system was smaller than in batch mode, which could be enhanced with further optimization of flow conditions. Regarding the STY values all of FeTPPS-based systems were much more effective then microsomal oxidations. Immobilized FeTPPS, applying a packed-bed continuous-flow reactor, was significantly more productive than classic shaked vial (batch) systems.

## 4. Discussion

The rise of continuous-flow systems in the field of preparative organic chemistry has given new impetus to the use of metalloporphyrin-based biomimetic systems, which have been widely used in the modeling of the oxidative metabolism of drugs since the 1990s, but have low efficiency in batch operation. Part of the official authorization (FDA, EMA) of drug candidates is the examination of the biological effect of metabolites formed in the human body, the prerequisite of which is the elucidation of the structure of the metabolites and their synthesis on a suitable scale. These prerequisites are particularly difficult to fulfill in the event that either in vivo or in vitro microsome-based systems have a reduced rate of drug metabolism and clearance. Chloroquine (CQ), chosen as a model drug, is a very good example for this case, which was supported by previous in vivo and in vitro microsome experiments that we also presented in this study. In this context, we investigated whether our metalloporphyrin-based biomimetic system, which can be integrated into a continuous-flow system as well, can be extended to the production of human metabolites of CQ. Our tests confirmed that the selected FeTPPS/*t*-BuOOH system is suitable for the production of primary dealkylation (DCQ-**M1**: desethyl-CQ, DDCQ-**M3**: didesethyl-CQ) metabolites of CQ. In addition, based on the mass change units obtained in LC-MS analysis as a minor biomimetic metabolite, we were able to identify the mono-oxidated *N*-oxide derivative of DCQ (**M4**), as well as its hydroxymethylated derivative (**M5**) associated with the radical activation of the methanol component of the reaction medium, as well as its aldehyde derivative (**M6**) corresponding to its oxidative dehydrogenation. It is also important to highlight that, identical to the human metabolism, the formation of primary dealkylation metabolites (DCQ, DDCQ) was more favorable in the biomimetic system, and the main biomimetic product was the major in vivo metabolite, DCQ. Modification of the length of the modified silica side chain, which ensures the attachment of the metalloporphyrin, significantly influenced the efficiency of the biomimetic transformation of CQ and the product profile of the biomimetic metabolites in both batch and continuous-flow systems. Increasing the length of the original aminopropyl side chain (Silica-Am-1) significantly enhanced the biomimetic activity. At the same time, the chain length increased by one ethylamino unit (Silica-Am-2) was more favorable than the further extension of the chain length (Silica-Am-3) regarding both substrate conversion and the formation of major and minor metabolites. We also managed to verify the correlation between metalloporphyrin activity and optimal anchoring chain length in a continuous-flow system. Finally, the metabolic conversion efficiency of chloroquine was evaluated using the turnover number (TON) and space time yield (STY) values. The obtained TON and STY values proved that both the batch homogeneous and immobilized heterogeneous metalloporphyrin-based biomimetic systems transform CQ into the corresponding primary metabolites by at least 3 orders of magnitude more efficiently than the in vitro microsomal systems did. Furthermore, the translation of the batch operation into a continuous-flow biomimetic system increased the biomimetic conversion efficiency of CQ expressed in TON and STY by an additional 1–2 orders of magnitude.

In summary, it can be concluded that using the rationally designed immobilization of metalloporphyrin-based system, not only the efficiency of the oxidative metabolic transition can be increased, but also the product profile of the formed metabolites can be modulated. Thus, the optimized metalloporphyrin-based biomimetic system integrated into a continuous-flow reactor can become an effective tool for drug development for the production of individual metabolites, satisfying not only the structure elucidation, but also the quantitative needs of pre-clinical in vivo studies.

## Figures and Tables

**Table 1:** Component analysis of human (HLM), rat (RLM) and mouse (MLM) liver microsomal-based metabolism of chloroquine. Each test was performed in triplicate, Stdr < 5%.

	Metabolite Profile		
	HLM	RLM	MLM
	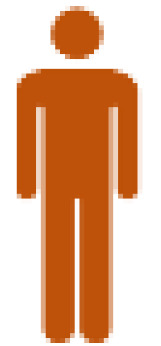	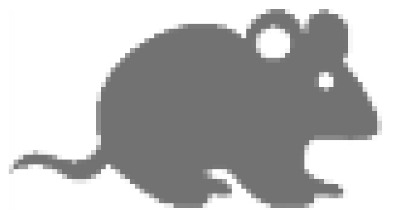	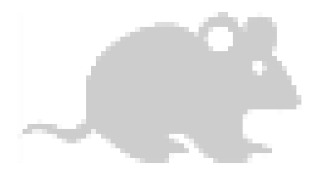
**Chloroquine** (CQ)	97.4	97.5	94.8
**M1** (CQ − 28u, −Et)	2.6	2.1	3.4
**M2** (CQ + 16, +O)	–	0.5	1.8
**c^1^ (%)**	2.6	2.5	5.2

c^1^ (%): conversion, all data represent UV peak area% (relative intensity at 220 ± 4 nm (see in detailed SI: Figures S1–S3. **M1**: N-desethyl-chloroquine (DCQ), **M2**: based on previous in vitro microsomal and in vivo studies, the mono-oxidation metabolite can probably be identified as a derivative of chloroquine N-oxide.

**Table 2:** Component analysis of dissolved FeTPPS and immobilzed FeTPPS metalloporphyin onto amino-functionalized silica particles (Silica-Am-1-FeTPPS, Silica-Am-2-FeTPPS and Silica-Am-3-FeTPPS) catalyzed biomimetic oxidation of chloroquine in batch mode. Each test was performed in triplicate, Stdr < 5%.

	Metabolite Profile (%)			
	FeTPPS	Silica-Am-1-FeTPPS	Silica-Am-2-FeTPPS	Silica-Am-3-FeTPPS
	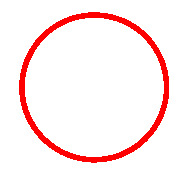	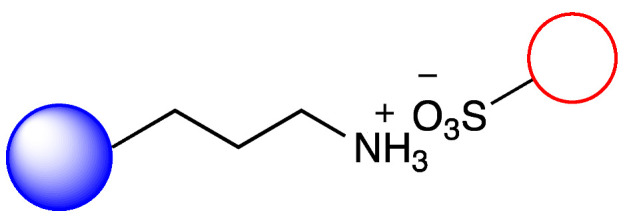	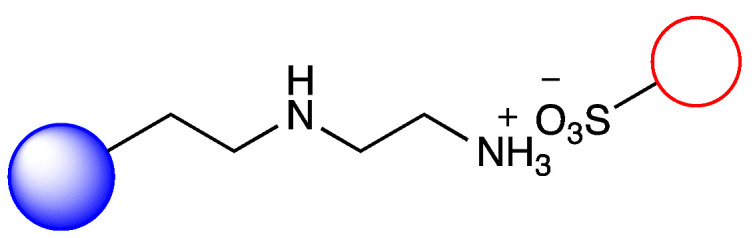	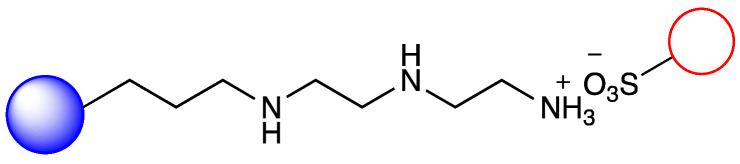
**CQ**	60.5	47.4	73.6	81.4
**M1** (CQ − 28u)	28.4	39.6	19.8	13.8
**M2** (CQ + 16)	–	–	–	–
**M3** (**M1** − 28u)	–	12.5	6.6	–
**M4** (**M3** + 16u)	0.2	0.2	0.1	<0.1
**M5** (**M1** + 30u)	0.1	–	–	<0.1
**M6** (**M5** − 2u)	0.2	0.1	–	–
**Other**	0.4	0.4	–	–
**c^1^ (%)**	39.5	52.6	26.4	18.6

c^1^ (%): conversion, all data represent UV peak area% (relative intensity at 220 ± 4 nm (see in detailed SI: Figures S4–S7. **M1**: N-desethyl-chloroquine (DCQ), **M2**: CQ N-oxide, **M3**: N-didesethyl-chloroquine (DDCQ), **M4**: mono-oxidation derivative of **M3**, **M5**: hydroxymethylated product of **M1**, **M6**: oxidative dehydrogenation of **M5**.

**Table 3:** Component analysis of immobilzed FeTPPS metalloporphyin onto amino-functionalized silica particles (Silica-Am-1-FeTPPS, Silica-Am-2-FeTPPS and Silica-Am-3-FeTPPS) catalyzed biomimetic oxidation of chloroquine (CQ) in continuos-flow mode. Tests were performed in triplicate, Stdr < 5%.

	Silica-Am-1-FeTPPS	Silica-Am-2-FeTPPS	Silica-Am-3-FeTPPS
	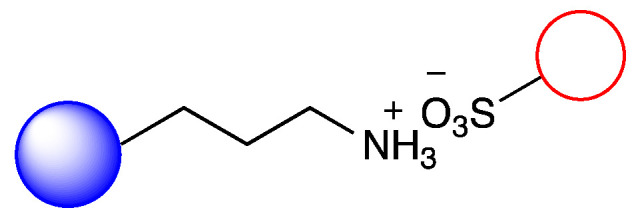	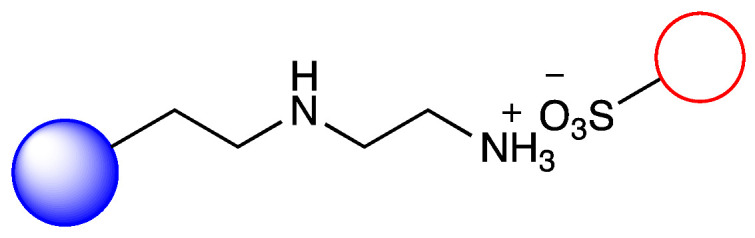	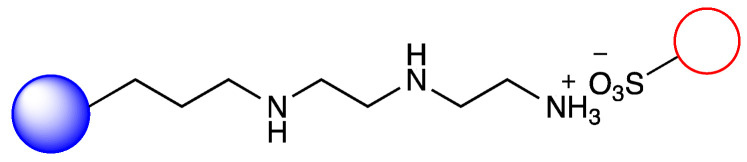
**CQ**	98.0	23.6	49.6
**M1 (DCQ)**	2.0	29.3	29.9
**M3 (DDCQ)**	–	36.6	19.3
**Other**	–	10.4	2.1
**c^1^ (%)**	2.0	76.4	50.4

c^1^ (%): conversion, all data represent UV peak area% (relative intensity at 220 ± 4 nm (see in detailed SI: Figures S8–S10, **M1**: N-desethyl-chloroquine (DCQ), **M3**: N-didesethyl-chloroquine (DDCQ).

**Table 4:** Comparison of different liver microsomal (HLM: human liver microsome, RLM: rat liver microsome, MLM: mouse liver microsome) and biomimetic system (dissolved FeTPPS and immobilized FeTPPS on silica particles: Silica-Am-1-FeTPPS, Silica-Am-2-FeTPPS and Silica-Am-3-FeTPPS) in the oxidative metabolism of chloroquine based on TON (Turn Over Number) and STY (Space Time Yield) values.

Catalyst	Reaction Mode	TON (–)	STY (mg L^−1^ h^−1^)
HLM	batch	–	0.32
RLM		–	0.31
MLM		–	0.64
FeTPPS		6.56	184
Silica-Am-1-FeTPPS	batch	8.73	296
Silica-Am-2-FeTPPS		4.38	119
Silica-Am-3-FeTPPS		3.09	84
Silica-Am-1-FeTPPS	continuous-flow	0.04	2309
Silica-Am-2-FeTPPS		1.62	88,213
Silica-Am-3-FeTPPS		1.07	58,193

## Data Availability

Not applicable.

## Supplementary Materials

The following supporting information can be downloaded at: https://www.mdpi.com/article/10.3390/metabo12121269/s1, Figure S1: Representative HPLC-DAD chromatograms of microsomal investigation of chloroquine (CQ) applying (a) human, (b) mouse and (c) rat liver microsome. Figure S2: Representative HPLC-DAD chromatograms of biomimetic oxidation of chloroquine (CQ) catalyzed by dissolved FeTPPS in batch mode. Figure S3: Representative HPLC-DAD chromatograms of biomimetic oxidation of chloroquine (CQ) catalyzed by FeTPPS immobilized on (a) Silica-Am-1 (b) Silica-Am-2 and (c) Silica-Am-3 in batch mode. Figure S4: Representative HPLC-DAD chromatograms of biomimetic oxidation of chloroquine (CQ) catalyzed by FeTPPS immobilized on (a) Silica-Am-1 (b) Silica-Am-2 and (c) Silica-Am-3 in continuous-flow mode. Figure S5: MS spectra of chloroquine (CQ). Figure S6: MS spectra of **M1** metabolite, desethyl-chloroquine (DCQ). Figure S7: MS spectra of **M2** metabolite, mono-oxidized metabolite of CQ. Figure S8: MS spectra of **M3** metabolite, didesethyl-chloroquine (DDCQ). Figure S9: MS spectra of **M4** metabolite, mono-oxidized metabolite of **M3**. Figure S10: MS spectra of **M5** metabolite, oxidative hydroxymethylated derivative of **M1**. Figure S11: MS spectra of **M6** metabolite, oxidative dehydrogenated derivative of **M5**.
