# Supplementary material for: Effect of Binding Linkers on the Efficiency and Metabolite Profile of Biomimetic Reactions Catalyzed by Immobilized Metalloporphyrin
Source: Metabolites. 2022 Dec 15;12(12):1269. doi: 10.3390/metabo12121269 (PMC9783926; doi:10.3390/metabo12121269)
Supplement: Supplementary file 1 [file metabolites-12-01269-s001.zip › metabolites-1995532-supplementary.pdf]

Effect of binding linkers on the efficiency and metabolite profile of biomimetic reactions catalyzed by immobilized metalloporphyrin

György T. Balogh [1,2,*], Balázs Decsi [3], Réka Krammer [3], Balázs Kenéz [3], Ferenc Ender [4,5], Tamás Hergert [6] and Diána Balogh-Weiser [3,7,*]

1 Department of Chemical and Environmental Process Engineering, Budapest University of Technology and Economics, Műegyetem rkp. 3., Budapest H-1111, Hungary
2 Institute of Pharmacodynamics and Biopharmacy, Faculty of Pharmacy, University of Szeged, Eötvös u. 6, H-6720 Szeged, Hungary
3 Department of Organic Chemistry and Technology, Budapest University of Technology and Economics, Műegyetem rkp. 3., Budapest H-1111, Hungary
4 SpinSplit Llc., Vend u. 17., Budapest H-1025, Hungary
5 Department of Electron Devices, Budapest University of Technology and Economics, Műegyetem rkp. 3., Budapest H-1111, Hungary
6 ThalesNano Ltd, Graphisoft Park, Záhony Str. 7, Budapest H-1031, Hungary
7 Department of Physical Chemistry and Materials Science, Budapest University of Technology and Economics, Műegyetem rkp. 3., Budapest H-1111, Hungary

* Correspondence: balogh.weiser.diana@vbk.bme.hu (D.B-W.), balogh.gyorgy@vbk.bme.hu (G.T.B); phone.: +3614632174

1. HPLC-DAD-MS chromatograms of microsomal investigation of chloroquine (CQ)

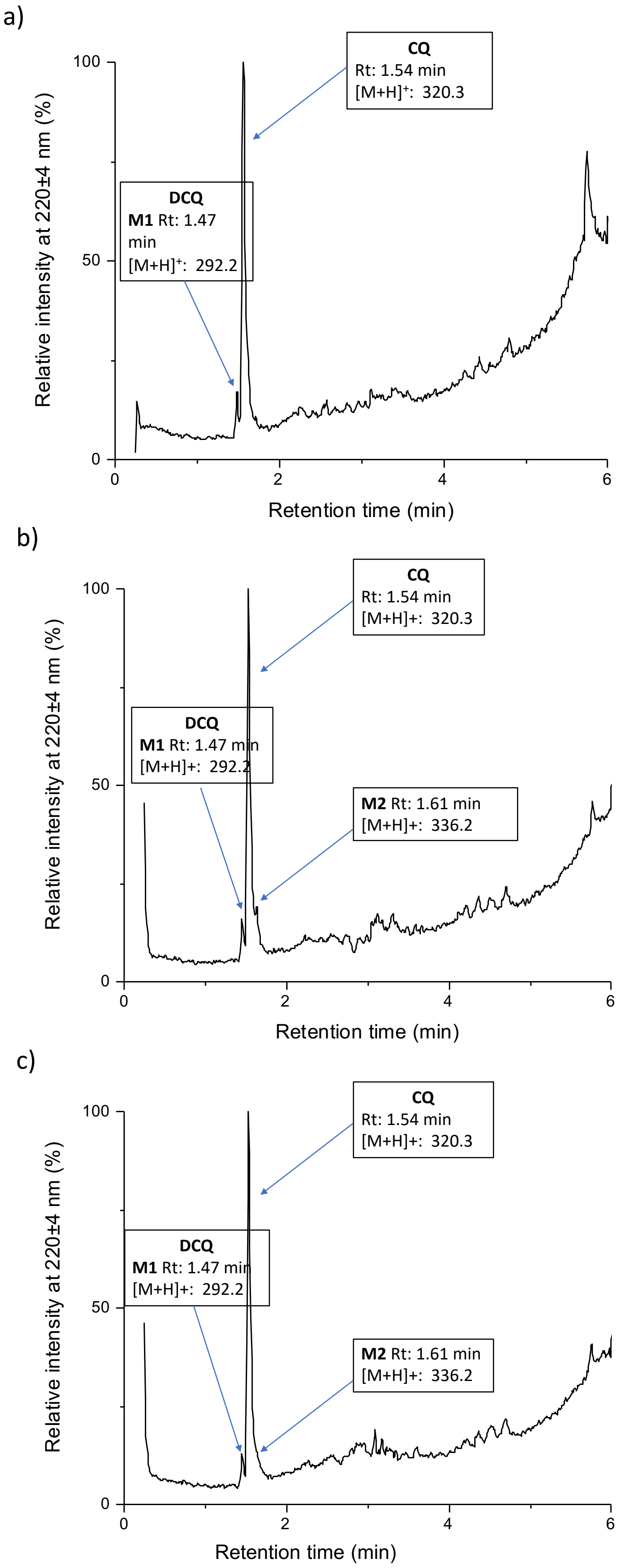

Figure S1. Representative HPLC-DAD chromatograms of microsomal investigation of chloroquine (CQ) applying a) human, b) mouse and c) rat liver microsome.

2. HPLC-DAD-MS chromatograms of FeTPPS catalyzed biomimetic oxidations of chloroquine (CQ)

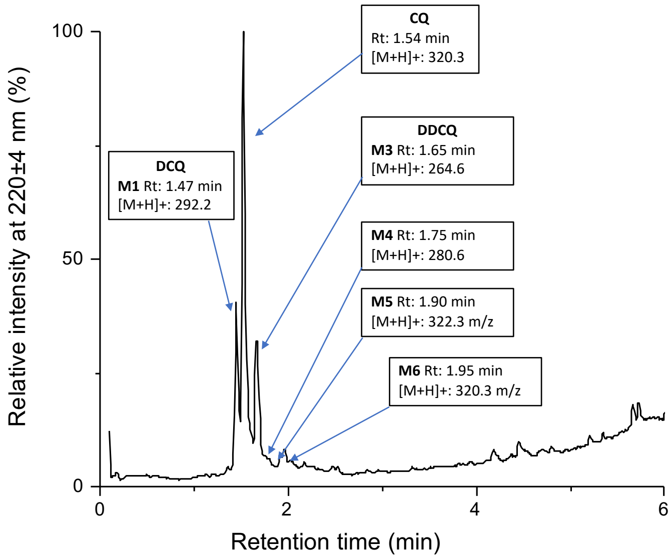

Figure S2. Representative HPLC-DAD chromatograms of biomimetic oxidation of chloroquine (CQ) catalyzed by dissolved FeTPPS in batch mode.

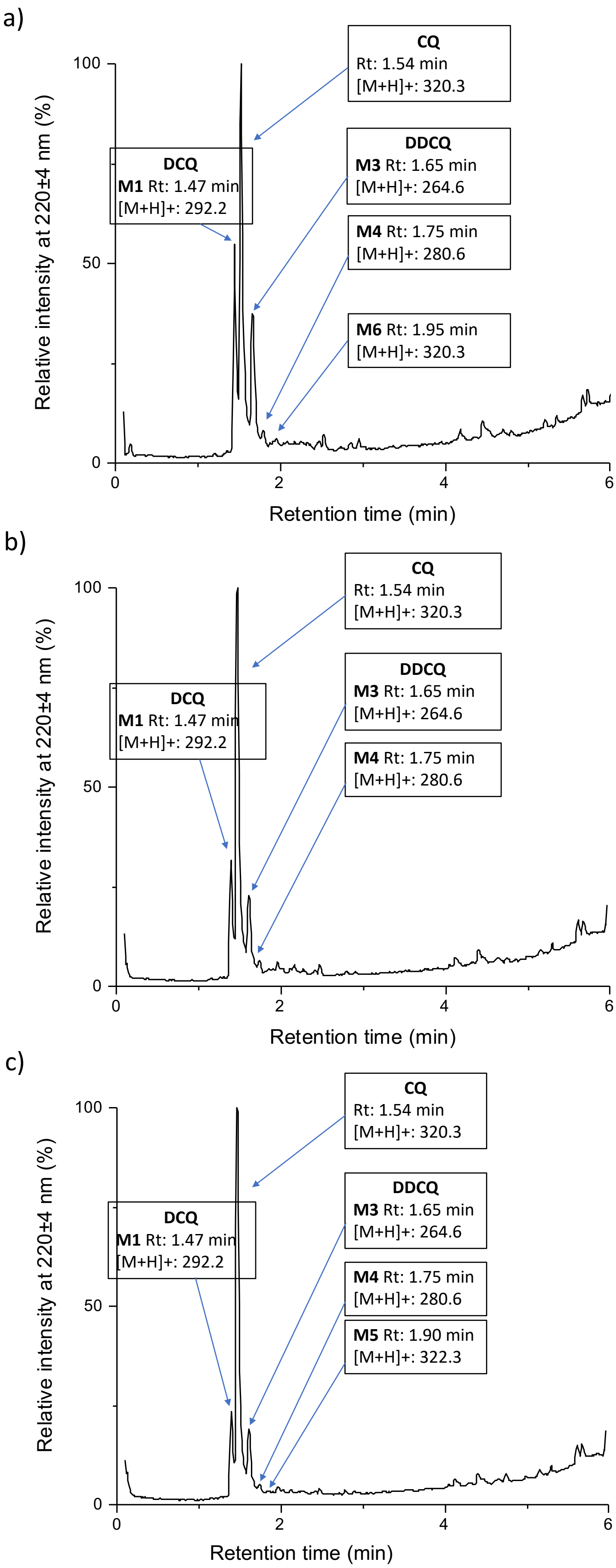

Figure S3. Representative HPLC-DAD chromatograms of biomimetic oxidation of chloroquine (CQ) catalyzed by FeTPPS immobilized on a) Silica-Am-1 b) Silica-Am-2 and c) Silica-Am-3 in batch mode.

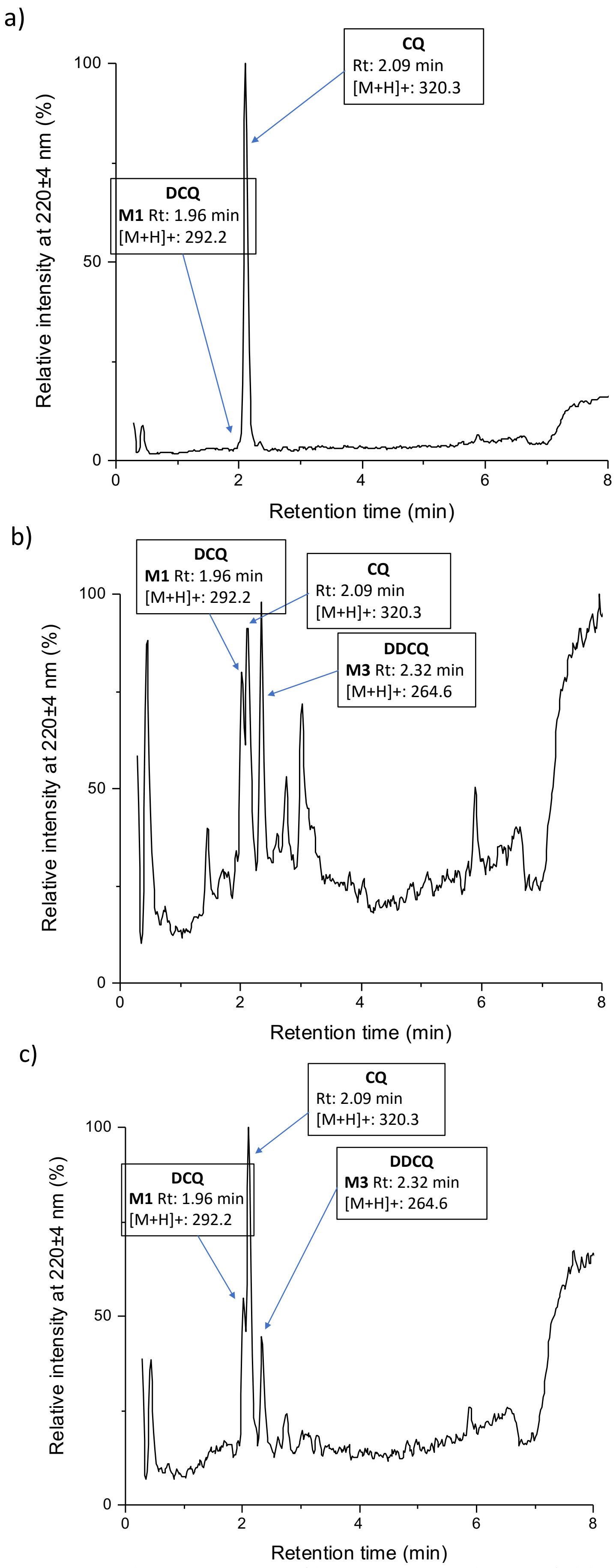

Figure S4. Representative HPLC-DAD chromatograms of biomimetic oxidation of chloroquine (CQ) catalyzed by FeTPPS immobilized on a) Silica-Am-1 b) Silica-Am-2 and c) Silica-Am-3 in continuous-flow mode.

3. MS Spectra of chloroquine (CQ) and its metabolites

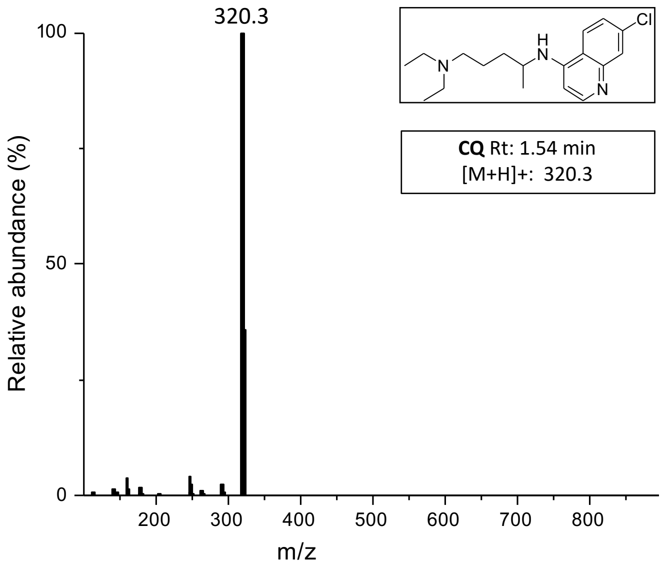

Figure S5. MS spectra of chloroquine.
(CQ)

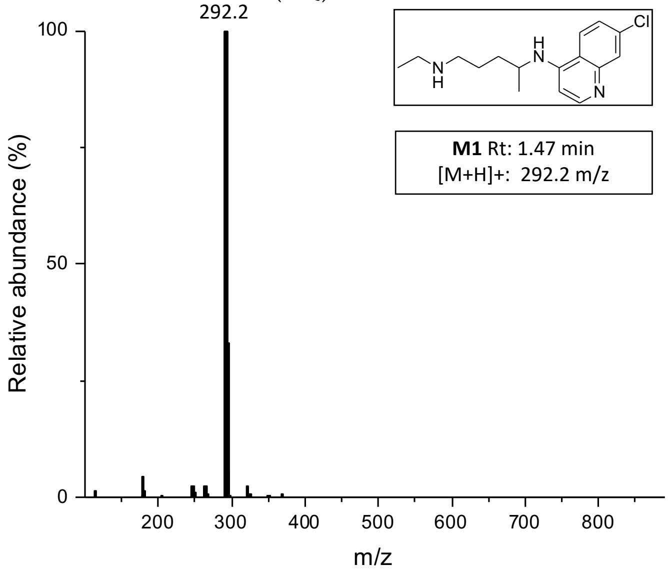

Figure S6. MS spectra of **M1** metabolite, desethyl-chloroquine (DCQ).

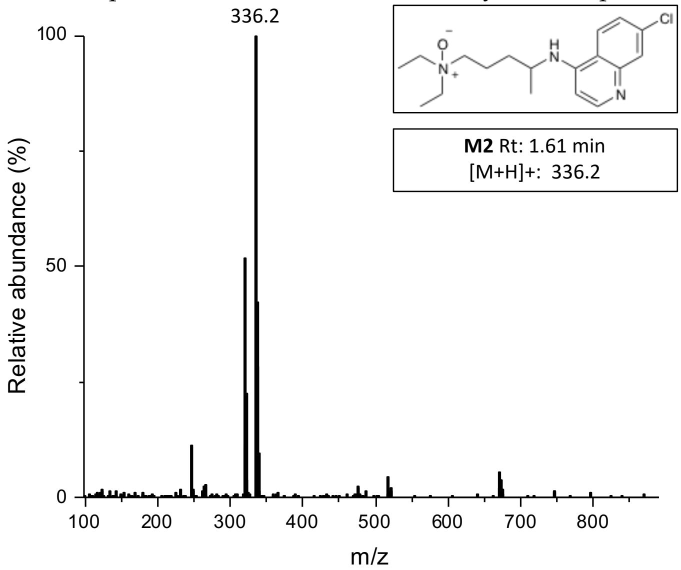

Figure S7. MS spectra of **M2** metabolite, mono-oxidized metabolite of CQ.

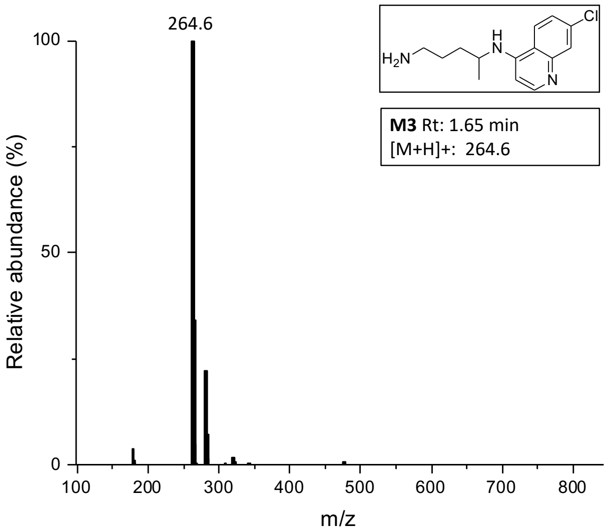

Figure S8. MS spectra of **M3** metabolite, didesethyl-chloroquine (DDCQ).

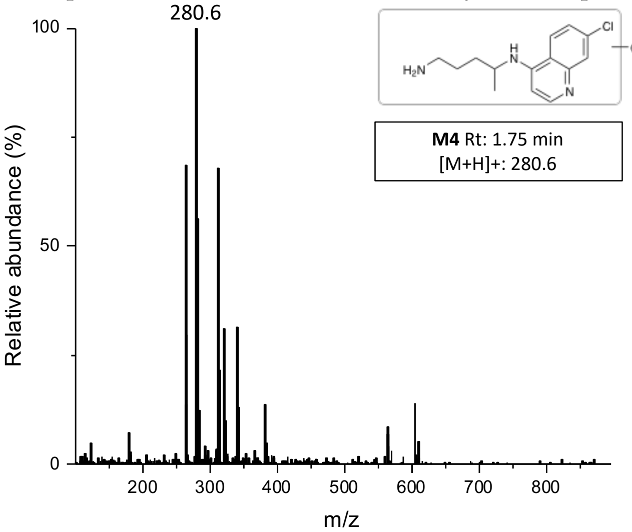

Figure S9. MS spectra of **M4** metabolite, mono-oxidized metabolite of **M3**.

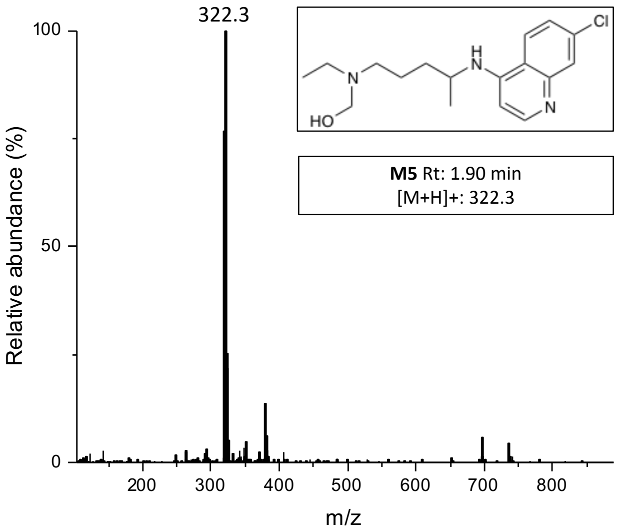

Figure S10. MS spectra of **M5** metabolite, oxidative hydroxymethylated derivative of **M1**.

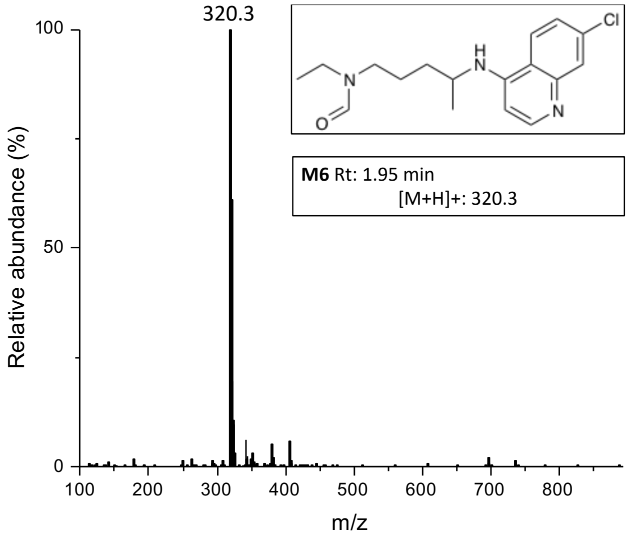

Figure S11. MS spectra of **M6** metabolite, oxidative dehydrogenated derivative of **M5**.
